# Multi-Systemic Alterations by Chronic Exposure to a Low Dose of Bisphenol A in Drinking Water: Effects on Inflammation and NAD^+^-Dependent Deacetylase Sirtuin1 in Lactating and Weaned Rats

**DOI:** 10.3390/ijms22189666

**Published:** 2021-09-07

**Authors:** Antonietta Santoro, Marika Scafuro, Jacopo Troisi, Giuseppe Piegari, Paola Di Pietro, Elena Mele, Donato Cappetta, Marianna Marino, Antonella De Angelis, Carmine Vecchione, Orlando Paciello, Silvia Fasano, Riccardo Pierantoni, Andrea Viggiano, Rosaria Meccariello

**Affiliations:** 1 Department of Medicine, Surgery and Dentistry “Scuola Medica Salernitana”, University of Salerno, 84081 Baronissi, Italy; ansantoro@unisa.it (A.S.); troisi@theoreosrl.com (J.T.); pdipietro@unisa.it (P.D.P.); mamarino@unisa.it (M.M.); cvecchione@unisa.it (C.V.); aviggiano@unisa.it (A.V.); 2 Department of Precision Medicine, University of Campania “Luigi Vanvitelli”, 80138 Naples, Italy; marika.scafuro@unicampania.it; 3 Theoreo srl Spinoff Company of the University of Salerno European Biomedical, 84090 Montecorvino Pugliano, Italy; 4 Unit of Pathology, Department of Veterinary Medicine and Animal Production, University of Naples Federico II, 80137 Naples, Italy; giuseppe.piegari@unina.it (G.P.); paciello@unina.it (O.P.); 5 Department of Movement Sciences and Wellbeing, Parthenope University of Naples, 80133 Naples, Italy; elena.mele@collaboratore.uniparthenope.it; 6 Department of Experimental Medicine, University of Campania “Luigi Vanvitelli”, 80138 Naples, Italy; donato.cappetta@unicampania.it (D.C.); antonella.deangelis@unicampania.it (A.D.A.); silvia.fasano@unicampania.it (S.F.); riccardo.pierantoni@unicampania.it (R.P.); 7 IRCCS Neuromed, Department of Vascular Physiopathology, 86077 Pozzilli, Italy

**Keywords:** BPA, liver, cerebral ventricles, Sirt1, *Sirt1-AS LncRNA*, epigenetics, inflammation

## Abstract

Bisphenol A (BPA) is largely used as a monomer in some types of plastics. It accumulates in tissues and fluids and is able to bypass the placental barrier, affecting various organs and systems. Due to huge developmental processes, children, foetuses, and neonates could be more sensitive to BPA-induced toxicity. To investigate the multi-systemic effects of chronic exposure to a low BPA dose (100 μg/L), pregnant Wistar rats were exposed to BPA in drinking water during gestation and lactation. At weaning, newborn rats received the same treatments as dams until sex maturation. Free and conjugated BPA levels were measured in plasma and adipose tissue; the size of cerebral ventricles was analysed in the brain; morpho-functional and molecular analyses were carried out in the liver with a focus on the expression of inflammatory cytokines and Sirtuin 1 (Sirt1). Higher BPA levels were found in plasma and adipose tissue from BPA treated pups (17 PND) but not in weaned animals. Lateral cerebral ventricles were significantly enlarged in lactating and weaned BPA-exposed animals. In addition, apart from microvesicular steatosis, liver morphology did not exhibit any statistically significant difference for morphological signs of inflammation, hypertrophy, or macrovesicular steatosis, but the expression of inflammatory cytokines, Sirt1, its natural antisense long non-coding RNA (*Sirt1-AS LncRNA*) and histone deacetylase 1 (Hdac1) were affected in exposed animals. In conclusion, chronic exposure to a low BPA dose could increase the risk for disease in adult life as a consequence of higher BPA circulating levels and accumulation in adipose tissue during the neonatal period.

## 1. Introduction

Bisphenol A [BPA,2,2-bis (4-hydroxyphenyl) propane] is one of the most common environmental pollutants routinely used to produce epoxy resins and polycarbonate plastics [1,2]. Acidic/basic food and drinks, high temperature or biological fluids cause BPA leaching in the environment and humans are commonly exposed through skin contact, inhalation, or ingestion of contaminated foods and beverages [3]. BPA can interfere with the endocrine system through mechanisms involving the link to steroid receptors like the nuclear estrogen receptor (ER) (i.e., ERα and ERβ), the membrane estrogen receptor GPER30, the estrogen-related receptor γ (ERRγ), or androgen receptor (AR), thus exhibiting estrogenic and anti-androgenic activities [4]. Therefore, BPA causes reproductive, developmental, inflammatory, and metabolic dysfunctions in humans and animal models [2,3,5,6,7,8,9,10].

When ingested, BPA’s fate and disposition in the body first depend on the ability of the gastrointestinal tract and liver to metabolise the compound to a hydrophilic form with low estrogenic activity. Glucuronidation by UDP-glucuronosyltransferases (UGTs) is the main pathway of BPA metabolic detoxification in both the intestine and liver [11]. In the proximal intestine, the transformation of unconjugated BPA (free BPA) into conjugated BPA (BPA-G) limits the adsorption of free BPA, thus representing a barrier that prevents BPA toxic effects. In the liver, free BPA is thought to be rapidly glucuronidated in the hepatic microsomes to be excreted through bile or urine as BPA-G. However, tissues like the intestine, liver, kidney, or placenta express the enzyme *β*-glucuronidase that can deconjugate BPA and thus release its active form again [11]. Consequently, BPA bio-accumulates in biological tissues and fluids like white adipose tissue [12] and maternal milk [13,14] and bypasses the placental barrier, thus reaching the foetus [15,16,17]. In animal models and humans, the BPA-dependent outcomes on health depend on life stage, doses, exposure route or time as well, but gestational, neonatal and perinatal timeframes are more sensitive to BPA exposure due to massive developmental processes [1] and the limited ability of the liver to conjugate BPA with respect to adult life [11]. Nevertheless, while oxidative stress and tissue damages have been considered for a long time as the main consequences of BPA exposure, recently, epigenetic effects—including the transgenerational ones—leading to behavioural, reproductive, and metabolic disorders have been documented [3,5].

In the brain, BPA interferes with the central control of reproduction [5] and also in energy homeostasis, appetite regulation [18,19], and immune surveillance functions [20], with activity on the hypothalamic reproductive neurohormones, orexigenic/anorexigenic neuropeptides, microglia, and astrocytes [5,9,10,20]. Apart from the effects on gamete quality, reproduction, and fertility [3,5], at the periphery, BPA targets both liver and white adipose tissue causing the deregulation of glucose and lipid metabolism and affects adipogenesis [9]. Thus, the main consequence of BPA exposure in the liver is the occurrence of oxidative stress, impaired mitochondrial functions, and increased lipid storage, ultimately leading to liver steatosis [9,21,22,23,24]. Epigenetic reprogramming of genes involved in lipid metabolism has been demonstrated in the liver of long-term exposed mice [25]. In this respect, BPA could act as an obesogenic compound, raising the risk of overweight and obesity in both exposed subjects and their offspring through trans-generational epigenetic mechanisms [19,26].

Interestingly, the Sirtuin 1 (Sirt1), the nuclear NAD^+^-dependent class III deacetylase, is an epigenetic target of environmental exposure to toxicants [27], being directly involved in coupling the cellular metabolic status (via NAD^+^) to the modulation of both chromatin structure and gene expression with mechanisms involving the deacetylation of histone proteins or non-histone proteins like transcription factors, co-factors, or nuclear receptors (i.e., FOXO-1, p53, Nf-kb, PGC1-α, AR, ERα, LXRa, etc.) [28,29]. Sirt1, also known as the “longevity gene”, protects cells against apoptosis and inflammation and is involved in the control of cell proliferation, differentiation, or death and has gained considerable attention in epigenetic mechanisms [30]. Changes in Sirt1 expression are critical in several diseases, such as cardiovascular diseases, metabolic syndrome, cancer, neurodegeneration, and infertility [29,31,32,33,34,35]. In the liver, Sirt1 is involved in the control of both glucose and lipid metabolism [36,37] with different downstream pathways, including histone modification in the nucleus or p53 dependent apoptotic pathways aimed at counteracting the accumulation of hepatic oxidative stress and inflammation [37].

Since BPA exposure may represent a health risk, in this manuscript, we analysed the multi-systemic alterations in rat tissues induced by chronic exposure to a low dose of BPA. In order to mimic one of the main exposure routes in humans, BPA was administered in drinking water first to dams during pregnancy and lactation and then, at weaning, also to the newborns. Hence, free BPA and BPA-G were measured in plasma, and BPA accumulation was measured in white adipose tissue. Then, morpho-functional evaluations were carried out in the brain and liver because they are well-known targets of BPA activity. In parallel, the expression profile of molecular markers of inflammation (i.e., inflammatory cytokines and the inflammation related transcriptional factor Nf-kb) and Sirt1 protein were compared in the livers of lactating and weaned animals. To provide further insights into the possible epigenetic targets of BPA, *Sirt1* mRNA, its natural antisense long non-coding RNA (*Sirt1-AS LncRNA*), and the expression rate of the main DNA methylation and deacetylating enzymes were also analysed in the livers of weaned animals.

## 2. Results

### 2.1. Experimental Groups and Body Weight

The experimental groups were composed as described in Table 1, the mean body weight did not differ between control and BPA treated animals at all ages and in both sexes.

### 2.2. BPA Concentration in Plasma and Adipose Tissue

**Figure 1:**
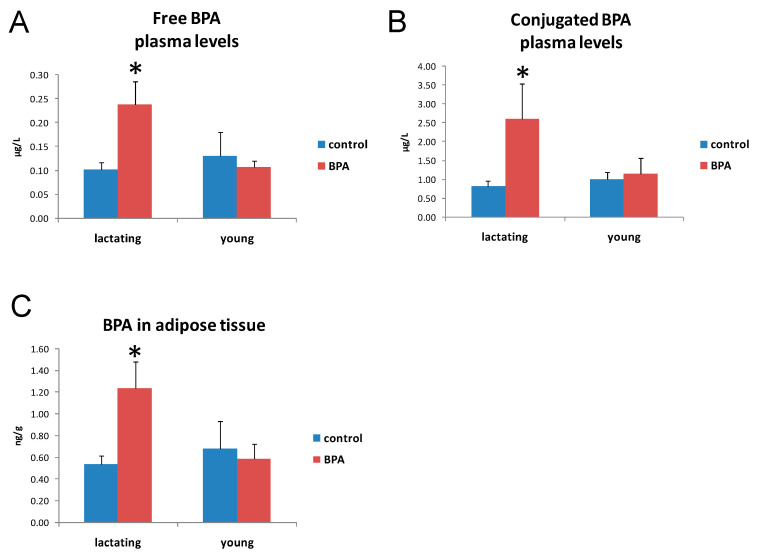
Free-BPA plasma levels in lactating and young rats drinking BPA-added (BPA) or tap water (control) (**A**). Conjugated-BPA plasma levels in lactating and young rats drinking BPA-added (BPA) or tap water (control) (**B**). BPA levels in adipose tissue from lactating and young rats drinking BPA-added (BPA) or tap water (control) (**C**). Data are reported as mean and SE. * *p* < 0.05 compared to all other groups.

BPA exposure produced an increased concentration of free-plasma-BPA (Figure 1A), plasma-BPA-G (Figure 1B), and adipose-tissue-BPA (Figure 1C) in lactating but not in young rats.

The ANOVA for free-plasma-BPA showed a significant effect for the treatment (F1,24 = 5.49, *p* < 0.05), for time (F1,24 = 4.7, *p* < 0.05), and for the treatment x time interaction (F1,24 = 8.23, *p* < 0.01). The Tukey’s post hoc test demonstrated that the BPA-treated group at 10–17 PND was statistically different from the others (*p* < 0.05).

The ANOVA for BPA in adipose tissue showed a significant effect dependent on the treatment (F1,24 = 4.41, *p* < 0.05), the time (F1,24 = 6.44, *p* < 0.05), and the treatment x time interaction (F1,24 = 6.96, *p* < 0.05). The Tukey’s post hoc test demonstrated that the BPA-treated group at 10–17 PND was different from the others (*p* < 0.05).

### 2.3. Nissl Staining and Measurement of Ventricle Size

**Figure 2:**
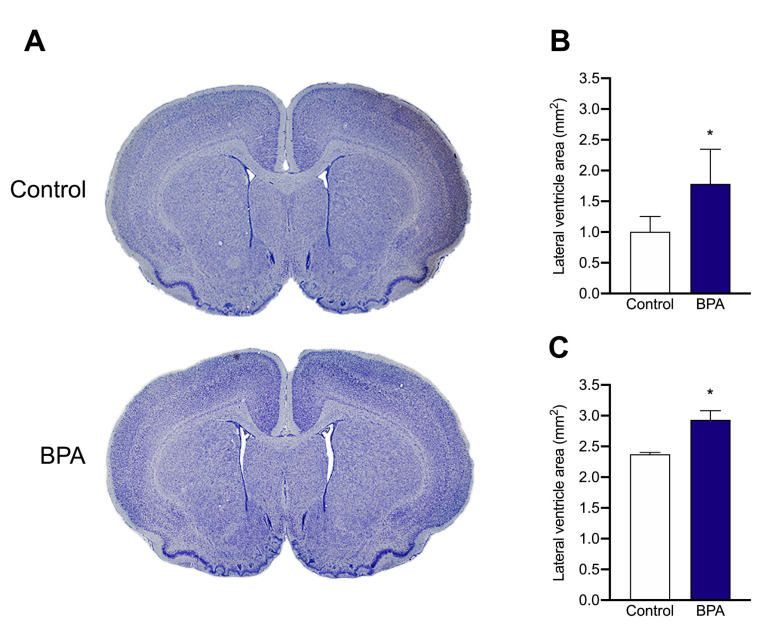
(**A**), Representative brain Nissl-stained sections of rats from PND 17 BPA-treated and control dams. (**B**,**C**), Bar graphs showing the lateral ventricular size measured by cross-sectional area of the brains in (**B**), PND 17 and (**C**), PND 60 rats from vehicle- and BPA-treated dams. Data are expressed as mean ± SE, *n* = 5 per group, * *p* < 0.05.

To assess the effects of BPA exposure in brain structures known to be important for the maintenance of neurons and glial homeostasis, we analysed lateral ventricles morphology and size on lactating and weaned rats (Figure 2A). Quantification of the total ventricular area revealed significant enlargement of the lateral ventricles in the brains of lactating and weaned rats chronically exposed to BPA (*p* < 0.05 vs. control group) (Figure 2B).

### 2.4. Histological Analysis of the Liver

**Figure 3:**
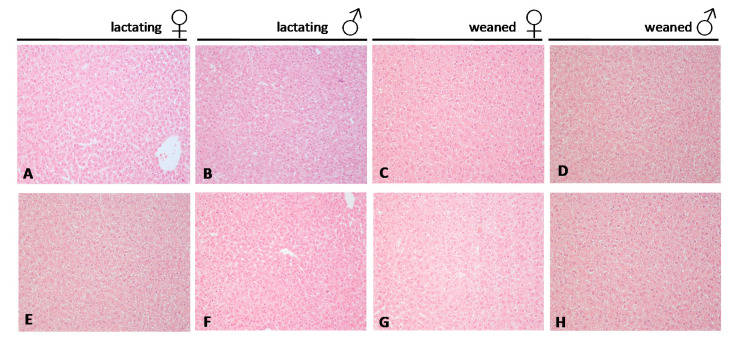
Representative haematoxylin and eosin–stained sections from liver tissue of rats. (**A**–**D**) controls, (**E**–**H**) BPA treated. (original magnification 20×). Females ♀, males ♂.

The main histological alteration of the liver was a mild to moderate microvesicular steatosis in all groups except in the control lactating group (Figure 3, Table 2). The Kruskal–Wallis test showed a significant difference for the microvesicular steatosis between the groups (K = 9.25, *p* < 0.05). Pairing comparison with the Mann–Whitney U test showed that the lactating control group was different from the others (Table 2). Mild hepatocellular hypertrophy was observed in some BPA exposed lactating or weaned animals. After grouping data from lactating and weaning rats, the Mann–Whitney U test showed a significant difference between the BPA-treated and the control groups.

### 2.5. Molecular Markers in Liver

#### 2.5.1. Inflammation

The expression rate of *Tnfα, Il-6,* and *Nf-kb* was analysed by qPCR in lactating and young animals by qPCR (Table 3). The expression rate of all the selected biomarkers resulted to be significantly higher in lactating BPA exposed animals (*p* < 0.05 vs. control group). *Tnfα* and *Nf-kb* mRNA remained higher in weaned BPA exposed animals, with weaker effects in lactating animals. *Il-6* mRNA levels did not change in weaned animals.

#### 2.5.2. Oxidative Stress

**Figure 4:**
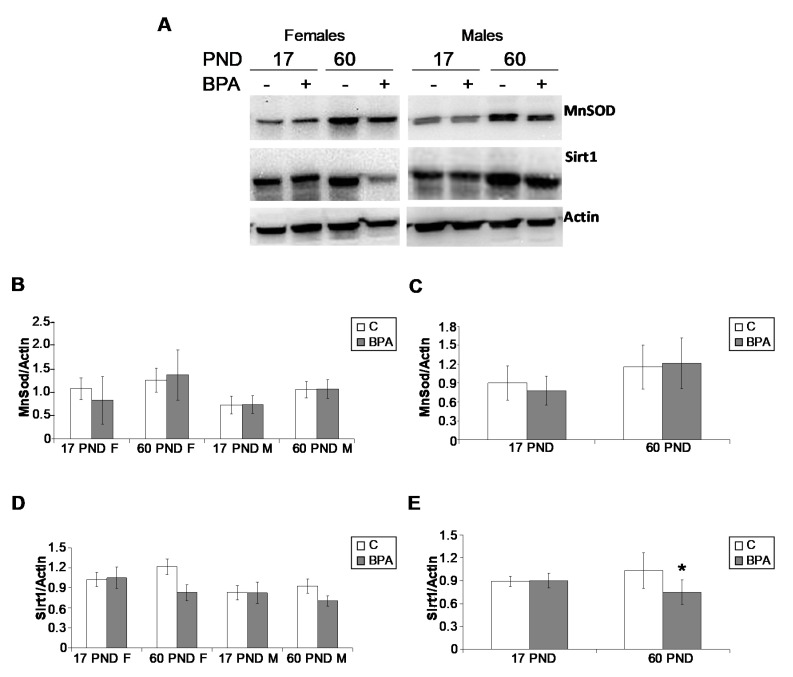
Representative Western blot for MnSOD and Sirt1 carried out in liver samples collected from lactating and young animals. (**A**). Data were normalised against actin–*β*, and are reported in (**B**–**E**) as mean value ± SD (*n* = 3 in B and D, *n* = 6 in C and E, 3 males and 3 females). F: females; M: males; PND: postnatal day. * *p* = 0.0143.

The mitochondrial manganese superoxide dismutase (MnSOD or SOD2), a key biomarker of oxidative stress, was analysed by Western blot (Figure 4). The enzyme expressed in both lactating and young animals did not show any statistically significant difference related to age, sex, or treatment.

#### 2.5.3. Epigenetics and Epigenetic Machinery

Sirt1 protein was analysed by Western blot in lactating and young animals (Figure 4). The protein resulted in being constantly expressed in lactating animals (17 PND); however, a significant decrease was observed only in 60 PND BPA treated animals (*p* < 0.05).

**Figure 5:**
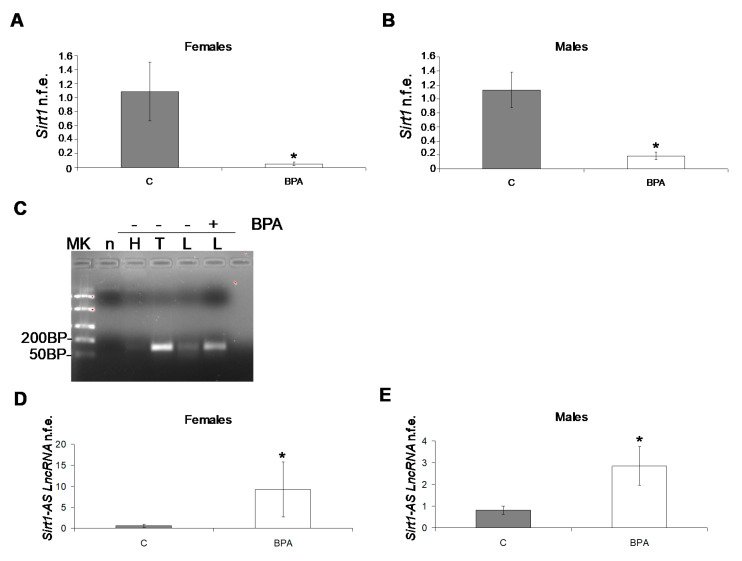
*Sirt1* qPCR in male (**A**) and female young animals (60 PND) (**B**). Strand-specific PCR for the detection of *Sirt1-AS LncRNA* in rat tissues (MK, DNA ladder; n, negative control; H, heart, T, testis; L, liver; -BPA absence; + BPA presence) (**C**). Quantitative PCR analysis for *Sirt1*-*AS LncRNA* in male (**D**) and female young animals (60 PND) (**E**). qPCR data are expressed as normalized fold expression (n.f.e.) ± SE, reference gene *Gapdh*; reference sample arbitrarily assigned value 1, control group (*n* = 6, 3 males and 3 females, * *p* < 0.05).

Therefore, the expression profile of *Sirt1* mRNA was further investigated at 60 PND by qPCR, confirming that *Sirt1* mRNA in BPA treated animals was significantly lower (*p* < 0.05) than the control group in both sexes (Figure 5A,B).

Since the evidence in the literature revealed the presence of *Sirt1 natural antisense long non-coding RNA* (*Sirt1-AS LncRNA*), an endogenous modulator of *Sirt1* mRNA stability in mouse [38,39] and human [40] rising from *Sirt1* gene 3′ end, *Sirt1* sequence was compared in rodents (mouse vs. rat, 97% (262/271) nucleotide identity in the gene region responsible for the formation of *Sirt1-AS LncRNA*. Hence, the possible presence of *Sirt1-AS LncRNA* was investigated in rat tissues [heart, positive control, and testis from control animals; liver from control and BPA exposed animals] by strand-specific PCR (Figure 5C). A band of the predicted size of 124 bp was detected in all tested tissues, particularly in the testis of control animals and in the liver of BPA exposed animals. Therefore, the expression rate of *Sirt1-AS LncRNA* was analysed by qPCR, revealing significantly higher expression in the liver of young animals, without any sex-dependent difference (Figure 5D,E).

In addition, the expression rate of the main enzymes involved in maintaining and de novo establishment of DNA methylation, *Dnmt1* and *Dnmt3a,* respectively, in parallel to histone deacetylases *Hdac1* and *Hdac2* were analysed by qPCR in young animals (Table 4).

*Dnmt1* and *Dnmt3a* mRNA levels were constant in young BPA exposed animals. However, the expression level *of Dnmt1* was slightly lower in young animals, but this difference was not statistically significant.

*Hdac1* mRNA was significantly higher in the treated animals, particularly in female rats. *Hdac2* expression level remained constant in young animals, but a different expression trend was observed in females and males.

#### 2.5.4. Lipid and Glucose Metabolism

The expression rate of *glucose-6-phosp*hatase (*G6Pase*), *Steroidogenic Acute Regulatory Protein* (*StAR*), and *Liver X receptor alpha* (*LXRa*) was analysed by qPCR and was constant in control and BPA exposed young weaned animals (Table 5).

## 3. Discussion

In recent years, the impact of BPA exposure on health has received more and more attention. This widely used environmental pollutant interferes in the homeostasis of the endocrine system causing developmental, reproductive, inflammatory, and metabolic dysfunction in humans and animals [2,3,5,6,7,8,9,10]. Epidemiological studies revealed significant urinary BPA concentration in large populations [41] and the correlation between BPA exposure and the development of chronic diseases [42,43], suggesting health risk. Nevertheless, BPA effects strongly depend on life stage, doses, exposure route, or time as well, with exposure in early life more deleterious than exposure in adult life [1,44]. Currently, its concrete risk for health is debated [45], and in several countries, its use has been restricted for some articles like infant feeding bottles [46]. The development of BPA substitutes like Bisphenol B (BPB), Bisphenol F (BPF), Bisphenol S (BPS), or Bisphenol AF (BPAF) did not help in solving BPA-related issues as preliminary studies have revealed these BPA analogues are not fully safe for health [14,47,48,49]. Therefore, additional studies on BPA mimicking human exposure routes (i.e., oral exposure by food or beverage) and doses currently considered “safe” for health are needed.

In the present study, we chronically exposed rats to BPA, first during the gestational and lactating phase through the administration of BPA in drinking water to dams; then, at weaning, the offspring were assigned to the same treatment groups as their mothers and started receiving BPA or vehicle in drinking water. Daily exposure dose of 10 μg/kg bw has been predicted accordingly to daily consumption of drinking water. Since the European Food Safety Authority (EFSA) has established tolerable daily intake (t-TDI) of 4 μg/kg bw-day, against the 50 μg/kg bw-day in the USA [50,51], the BPA dose used in this study is within the reference limit for humans, currently considered “safe” for health. We found circulating free and BPA-G levels in both lactating and weaned young animals, including control groups, probably as a consequence of general environmental exposure. However, both free and BPA-G levels resulted significantly higher than control groups only in animals exposed to BPA during lactation; similarly, lactation is the only exposure window with significant bio-accumulation of BPA in white adipose tissue. BPA bio-accumulates in maternal milk [13]. In infant rats and mice, the ability of the liver to conjugate BPA is limited, and in humans, BPA body burden decreases with age and intake rates are higher in children and adolescents than in adults [52]. In this respect, the lactation phase deserves particular attention for the health risk related to BPA exposure. In fact, maternal milk may represent an important vehicle for BPA transfer from mother to offspring in a time frame critical for the developmental process and possible disease load in later life. Thus, BPA has generated considerable concern, and in the present manuscript, the brain and liver were chosen as target tissues to evaluate the effects of BPA chronic exposure.

The developing brain is a particularly sensitive BPA target [5]. Present data reveals significant enlargement of the lateral ventricles in the brains of both lactating and weaned rats, suggesting BPA interference in postnatal brain development and brain functions in adult life. Consistently, in the same cohort of lactating animals used in this study, BPA exposure induced DNA damage in microglia and astrocytosis by decreasing ERα expression within the dentate gyrus, thus altering immune surveillance functions within the brain [20]. Ventricle enlargement may be the consequence of increased cerebrospinal fluid (CSF) pressure and circulation, especially in the “vulnerability window” of brain development [53]. The CSF maintains brain homeostasis, supplies nutrients and is an important source of biologically active molecules involved in stem cell proliferation, brain development, differentiation, and repair [54,55]. In this respect, CSF also functions as a lymphatic system for the brain, and CSF compartments are key to the immune surveillance of the Central Nervous System (CNS) [55,56]. Finally, CSF regulates intracranial pressure, playing an important role in volume transmission within the developing and adult brain [54]. Dysfunctions in the CSF circulation and in the size of cerebral ventricles are known to be linked to various pathologies such as hydrocephalus, encephalopathy, degenerative disease, and so on [57]. The significance of such a variation is still controversial, but some Authors have suggested that changes in the size and morphology of lateral ventricles may be related to subcortical white and/or grey matter alterations [57,58].

During pregnancy, BPA exposure may affect the health of both mother and offspring, causing excess body weight, insulin resistance, and hyper-insulinemia in the mothers and altered glucose homeostasis, increased weight, and adipogenesis in the offspring, which can display obese phenotype, glucose intolerance, and insulin resistance at adult age [44]. In the present study, the BPA exposed offspring did not display any significant changes in body weight gain during the lactating phase or as young animals. However, in 45 PND exposed animals, a significant increase in body weight was registered in male rats in relation to puberty and sex maturation [27]. Most histological features of the liver were normal over all the treatment without any significant differences related to age, sex, and diet (i.e., maternal milk vs. standard diet for weaned animals); mild microvesicular steatosis was present in all groups except in the control lactating group, and statically not significant hypertrophy was observed in all BPA exposed animals. Despite lacking macroscopical tissue alterations, at the molecular level, BPA is able to exert high stimulatory effects on the expression rate of inflammatory cytokines (i.e., *Tnfα* and *Il-6*) and *Nf-kb*-mediated inflammation in lactating exposed offspring. BPA effects on gene expression were still persistent, but on a lighter level, in weaned young animals with *Tnfα* and *Nf-kb* mRNA slightly over-expressed with respect to the control group and *Il-6* expressed at levels comparable to the corresponding control group. Taken together, our results suggest that the lactating period remains a critical exposure window, whereas a partial rescue can occur in young animals.

Notably, oxidative stress is the main consequence of BPA exposure [3,5,27], but, consistent with the morphological evaluation of the liver provided here, SOD2 protein, the main mitochondrial biomarkers of antioxidant defences against reactive oxygen species, was constantly expressed in the liver of lactating and young animals, confirming that the used BPA dose falls within the range of “safe dose” for health. By contrast, the metabolic sensor Sirt1 resulted in being a BPA target in both rat testis [27] and liver (present data). In the liver of young but not lactating exposed animals, Sirt1 protein resulted expressed at lower levels than control groups providing evidence for a possible predisposition to the occurrence of impairment in Sirt1-downstream pathways. In the liver, Sirt1 plays a beneficial role in regulating lipid metabolism and controlling oxidative stress and inflammation by deacetylating some transcriptional regulators against the progression of fatty liver disease [37]. *Sirt1* liver-specific knockout causes hepatic steatosis and promotes the progression to advanced metabolic disorders [37]. In our experimental model, micro-vesicular steatosis occurred in BPA-exposed lactating animals with the partial rescue of inflammation biomarkers at weaning. Sirt1 is a well-known suppressor of TNF*α*induced apoptosis [59]. Loss of Sirt1 activity regulates the expression of *Il-6* [60], and as reported above, these inflammatory cytokines resulted in being differently expressed in the liver of the BPA-treated animals collected at different ages. Interestingly, antagonistic crosstalk between Nf-kb and Sirt1 in the regulation of inflammation and metabolic disorders have been reported, with Sirt1 inhibition capable of disrupting oxidative energy metabolism and stimulating the Nf-kb-induced inflammatory responses that characterise several age-related and chronic metabolic diseases [61].

Natural antisense transcripts (NATs), originally considered as transcriptional noises arising from “junk DNA″, are recently recognised as important players in the epigenetic regulation of gene expression [62]. Recent studies demonstrated a self-modulatory loop in *Sirt1* activity involving its NAT, the *Sirt1-AS LncRNA.* This natural antisense long non-coding RNA is transcribed from the *Sirt1* gene antisense strand [38] and rescues *Sirt1* transcriptional suppression through the formation of an RNA-RNA duplex with the 3′ untranslated region of *Sirt1* mRNA [39]. In mice, this pathway inhibits *Sirt1* silencing mediated by *miR-34a* and promotes the stability of *Sirt1* mRNA during myogenic differentiation [39]; furthermore, it attenuates pulmonary fibrosis, enhancing the stability of *Sirt1* and increasing its expression during the epithelial-mesenchymal transition [63]. In humans, *Sirt1-AS LncRNA s*uppressed the miRNA-induced translational repression of *Sirt1* mRNA by masking the *miR-29c* binding site on the *Sirt1* 3′ untranslated region and promoting the proliferation of the human hepatocellular carcinoma (HCC) cell lines [40]. Furthermore, the *Sirt1* AS transcript was down-regulated in human tumours, while the *Sirt1* mRNA level was increased in cancer cell lines and cancer tissues [64]. In this paper, by specific stand PCR, we provide evidence of *Sirt1-AS LncRNA* in the heart (positive control), testis and liver of 60 PND rats. The decreased expression of *Sirt1* in the liver of BPA-exposed weaned rats (both females and males) parallels the increased expression rate of *Sirt1-AS LncRNA,* confirming the existence of a functional relationship between *Sirt1* mRNA and its AS transcript.

The epigenetic effects of BPA usually occur through changes in DNA methylation status, histone protein modifications, or the aberrant production of non-coding RNA [3]. Present data revealed that BPA treatment did not affect the expression rate of maintaining and *de novo* DNA methylation enzymes (i.e., *Dnmt1* and *Dnmt3a*), which are notably involved in the BPA dependent occurrence of insulin resistance, hepatic lipid accumulation, and steatosis in adulthood via aberrant methylation status in the promoter region of genes involved in glucose and lipid homeostasis [25,65,66]. Conversely, *HDAC1* mRNA resulted in being significantly higher in the exposed weaned animals. In this respect, the interaction between Sirt1 and other epigenetic markers/enzymes has been reported. Histones H1, H3, and H4, Dnmt1, and Hdac1 are well-known targets of Sirt1 [30], and during heat-shock stress, the acetylation of Hdac1 and the degradation of Sirt1 form a positive feedback loop to regulate p53 activity, one of the main downstream targets of Sirt1 related to apoptosis [67].

Thus in our system, a double route involving *Sirt1-AS LncRNA* and Hdac1 and aimed at compensating hepatic Sirt1 deficit may be suggested. Accordingly, the present study revealed that BPA exposed weaned animals did not exhibit any significant alterations in the expression rate of *G6Pase* and *LXRa-* targets of Sirt1protein [68,69], and *Star* that are involved in glucose homeostasis, bile acid synthesis, intracellular lipid homeostasis, cholesterol metabolism, and in the control of transcriptional programs critical for lipid homeostasis and inflammation. However, preliminary results from liquid chromatography-mass spectrometry and infrared spectroscopy limited to the cohort of 60 PND male rats used in this study revealed that the liver of sexually mature male rats chronically exposed to BPA had a different lipid and carbohydrate composition than the control [70], raising the possibility of metabolism-related disease load in later life. The decreased levels of Sirt1 (both mRNA and protein), a leader in the protective mechanisms against disease-related conditions, confirms the health risk of BPA exposure at low doses.

Thus, the present study gave evidence of the toxic effects of BPA from a low-dose drinking source, with particular emphasis on the risk during lactation and confirmed Sirt1 as a key epigenetic target of environmental exposure to toxicants. However, the present work did not evaluate a dose-response curve of BPA effects and did not obtain a direct measure of BPA levels in milk. These points could eventually be evaluated in future studies in parallel to the metabolic and neurological status of the older animals.

The interaction between diet quality and lean vs. overweight/obese phenotypes on BPA health effects is an additional interesting perspective to take into account. In fact, in animal models, BPA maternal nutrient supplementation counteracts BPA-induced DNA hypomethylation in early development, and dietary intervention, like the administration of antioxidants, could mitigate some of BPA’s adverse effects on health ([3,10] for review). Nevertheless, in humans, the association between overweight/obese phenotypes on BPA health effects is suggested [42,43] but still controversial due to the limited number of epidemiological and toxicological studies, sometimes revealing contradictory results. Therefore, additional studies are required in this field.

## 4. Materials and Methods

### 4.1. Chemicals and Antisera

BPA (Sigma Aldrich, Milan, Italy) was dissolved in ethanol (0.1 mg BPA/100 μL ethanol) and added to one litre of drinking water as previously reported [27]. In Western blot, primary antisera were: mouse monoclonal anti-Sirt1 diluted 1:5000 (ab110304; Abcam, Cambridge, MA, USA); anti-MnSOD diluted 1:1000 (06-984, Merck Millipore Milan, Italy); mouse monoclonal anti Actin-β diluted 1:2000 (A3853, Sigma Aldrich). Horse-radish conjugated secondary antisera (Santa Cruz Biotechnology Inc., Heidelberg, Germany) were used at 1:1000 dilution.

### 4.2. Animals and BPA Exposure Protocol

In vivo exposure of pregnant Wistar rats (*n* = 6) (Harlan Laboratories, Udine, Italy) was carried out as recently reported [37]. Each female was housed in a separate cage and was given in drinking water treatment [0.1 mg BPA dissolved in 100 μL ethanol for litre; BPA-exposed mothers, *n* = 3) or vehicle (100 μL/l ethanol; control mothers, *n* = 3)] by glass bottles. This treatment was expected to result in a daily BPA exposure of 10 µg/Kg b.w. for the treated group and 0 µg/Kg b.w. for the control group (considering an average daily water intake of 0.1 L/Kg b.w.). This treatment was maintained all the time also for the newborns, assigning each newborn to the same treatment group of the mother. Newborns were finally sacrificed at 10–17 postnatal day (PND) (BPA-exposed lactating group, *n* = 7 males, 6 females, and control lactating group, 6 males, 5 females) or at 45–60 PND (BPA-exposed young group, 10 males, 9 females, and control young group, 9 males, 7 females) following deep anaesthesia by an overdose of Tanax (0.1 mL intrapulmonary). Peripheral blood was collected, centrifuged to gain plasma, and stored at −80 °C until used for the measure of free BPA and BPA-G. Visceral adipose tissue and liver pieces were removed, quickly frozen in dry ice and stored at −80 °C until used for Reverse Transcriptase-quantitative Polymerase Chain Reaction (RT-qPCR), Western blot, or for BPA measure, as reported below. After that, animals were perfused with 4% paraformaldehyde (PFA), and fixed brain and liver pieces were collected and processed for histological evaluations. Hearts and testis were also collected from *n* = 3 vehicle-treated young animals (60 PND) and used as a positive control.

For the entire experiment, animals were housed under standard conditions with free access to fresh food with minimised levels of naturally-occurring phytoestrogens (Teklad Global Rodent Diets, Envigo, Italy) and tap water (with BPA added or vehicle as described) according to the EU norm for the use and care of experimental animals. The experimental protocol was approved by the Ethical Committees of the University of Salerno and by the Italian Ministry of Education, University and Research (authorisation number: 45/2014-PR 17 November 2014).

### 4.3. Free and Conjugate Plasma BPA Measure

BPA determinations were conducted as reported by Kosarac et al. [71], with some minor modifications as reported in [72,73]. Briefly, d16-BPA was added as an internal standard and samples were defatted with hexane and then extracted with dichloromethane. The extracts were purified in two successive SPE steps, one with a Florisil solid phase and one with a C18 solid phase. Deconjugation was carried out using β-Glucuronidase/Arylsulfatase from Helix pomatia (Sigma-Aldrich). After nitrogen drying, purified serum extracts were derivatised with N,O-Bis(trimethylsilyl)trifluoroacetamide (BSTFA) (Sigma-Aldrich). Derivatised samples were quantified using triple quadrupole mass spectrometer hyphenated with gas chromatography by means of GCMS TQ8030 (Shimadzu, Kyoto, Japan).

The gas chromatographic method was based on a SLB-5ms, 10 m × 0.1 mm, with a film thickness of 0.1 μm (Supelco, Milan, Italy). The GC oven program includes an initial phase of 1.5 min at 188 °C and two ramps: the first at 20 °C/min up to 260 °C, the second at 40 °C/min up to 320 °C. The injection temperature was set at 260 °C, and the linear velocity of the carrier gas (He) was 70 cm/s. The total analytical time was 8 min. Two acquisition channels were used, one in SCAN mode between *m*/*z* = 50 and *m*/*z* = 500 and one in MRM mode: 357.10 > 191.20 *m*/*z* for BPA and 370.50 > 73.10 *m*/*z* for the d16-BPA.

### 4.4. Adipose Tissue BPA Measure

An aliquot of 100 mg of adipose tissue from each sample was homogenised with 500 μL of water using an Ultra-Turrax^®^ (IKA^®^ T18 Basic) and spiked with d16-BPA as internal standard. Calibrant samples were prepared in adipose tissue by spiking tissue homogenate with a known volume of BPA solution to obtain final concentrations ranging from 0.1 ng/mL to 10.0 ng/mL. For calibration blanks and samples, fortifications were replaced with an aliquot of pure methanol. Five hundred microliters of acetonitrile were added to samples homogenates and then vortex-mixed for 1 min. A buffer solution containing magnesium sulphate, di and trisodium citrate, and sodium chloride was added, and pH was adjusted to 5.5 with NaOH 1 M. Solutions were vortex-mixed again for 1 min and centrifuged. The upper organic layer was added with 100 mg of diamino, primary secondary amine octadecyl modified silica phase end-capped (Machery-Nagel, Düeren, Germany), vortexed again for 30 s and centrifuged at 2500× *g* for 5 min. The purified upper organic layer was transferred into a clean glass tube and evaporated to dryness at 37 °C under a gentle nitrogen stream. Dry residues were derivatised and analysed as for serum samples.

### 4.5. Nissl Staining and Measurement of Ventricle Size

Brains (*n* = 5/group, 3 females and 2 males) were removed, post-fixed overnight in 4% PFA, and transferred in 70% ethanol until they were included in paraffin. Serial 10 µm coronal sections were sampled according to the atlas of Paxinos and Watson [74] and collected onto glass slides. Deparaffinised sections were then processed for staining with Nissl staining. Briefly, after rinsing in dH_2_O, sections were incubated for 8 min in thionin. The sections were then dehydrated through serial ethanol solutions (70 to 100%), cleared in xylene and mounted with Eukitt^®^ Quick-hardening mounting medium. Sections were imaged for bright-field microscopy using an upright microscope (Olympus BX53). Areas of lateral ventricle were manually traced from both sides, measured using ImageJ, and volumes were recorded in square millimetres.

### 4.6. Evaluation of Liver Morphology

PFA fixed livers were processed for paraffin embedding accordingly to standard procedures. Sections (5 µm) were mounted on slides and stained with haematoxylin and eosin for histological analysis. Histological features were evaluated using the General NAFLD Scoring System for Rodent Models [75] and were grouped into four broad categories: macrovesicular steatosis, microvesicular steatosis, hypertrophy, and inflammation. A modified Kleiner scoring [76] was used as summarised in Table 6.

### 4.7. Protein Extraction and Western Blot

The liver was homogenised with a dunce homogeniser at 4 °C in ice-cold RIPA buffer (3 mL/g tissue, added with protease inhibitor cocktail, all from Santa Cruz Biotechnology). After the addition of 300 μg of Phenylmethylsulfonyl fluoride (PMSF) per gram of tissue, lysate was kept in ice for 30′ and then centrifuged twice at 10,000× *g* for 10 min at 4 °C to gain total proteins extract in cleared supernatant. Protein concentration was then determined using the Lowry method [77].

Proteins (40 µg) were resolved by 8 or 12% sodium dodecyl sulfate-polyacrylamide gel (SDS-PAGE) and transferred into PVDF filters by Trans-Blot Turbo Transfer System (Bio-Rad Laboratories, Inc. Hercules, CA, USA). Filters were then stripped in Western blot stripping buffer (Santa Cruz Biotechnology Inc.) and reprobed with actin-β to normalise protein signals. Images were obtained by ChemiDoc-it 500 Imaging System (Bio-Rad Laboratories), and the optical density of the bands was analysed with Quantity One Analysis Software (Bio-Rad Laboratories). Data were expressed as mean protein/α-actin ratio ± SD.

### 4.8. Total RNA Extraction, Quantitative Reverse Transcription Polymerase Chain Reaction (qRT-PCR)

Total RNA was extracted from the liver, heart, and testis of weaned animals by RNA-XPress Reagent (Himedia, Mumbai, India) following the manufacturer’s instructions. Following DNaseI treatment (Thermo Fisher Scientific, Milan, Italy), 5 µg total RNA were reverse transcribed using a mixture of dT_18_/random examers by SuperScript-III RNase H^-^ Reverse Transcriptase (Thermo Fisher Scientific) in a total volume of 20 µL and following the instructions of the manufacturer.

All qRT-PCR assays were prepared in a final volume of 20 µL containing 1 µL of 1:5 diluted cDNA, 0.5 µM specific primer (details in Table 7), and 10 µL of SYBR Green Master Mix (Bio-Rad Laboratories). Each assay was carried out twice in duplicates in the Mastercycler CFX-96 (Bio-Rad Laboratories) and included melting curve analysis. The expression rate was normalised against *actin-β* or *Gapdh* by the ^ΔΔ^Ct method as previously described [78]. Data were then reported as mean fold change ± S.D. over the value one arbitrarily assigned to the control sample.

### 4.9. Evaluation of Sirt1-AS LncRNA: Strand Specific PCR and Expression Analysis

Following DNaseI treatment, 5 µg total RNA were reverse transcribed as reported in the previous paragraph substituting the mixture of dT_18_/random examers with 0.5 µM of the reverse primer of *Sirt1-AS lncRNA* (Table 7). Strand-specific RT-PCR was carried out using the 1 μL of diluted (1:5) cDNA, 10 pMol oligonucleotide primers (Table 7) in PCR mix [0.2 mM dNTP, 1× PCR buffer, 1.5 mM MgCl2, 1.25 U Taq Polymerase (Thermo Fisher Scientific)], using a DNA Engine ThermalCycler (Bio-Rad Laboratories) at the following conditions: 5 °C 30 s, 60 °C 30 s, 72 °C 30 s for 30 cycles; PCR products were checked on 2% agarose gel to reveal an amplificate of the predicted size of 124 bp.

### 4.10. Statistical Analysis

Data were expressed as mean ± standard error (SE) or standard deviation (SD). Student’s *t*-test was performed evaluating the differences between BPA exposed groups and controls. Comparisons between multiple groups were assessed by two-way ANOVA followed by Tukey’s post hoc test for parametric data or by the Kruskal–Wallis and the Mann–Whitney U tests for non-parametric data. Statistical analysis was performed with GraphPad Prism software (version 5.0, GraphPad Software, San Diego, CA, USA). A *p* < 0.05 was considered statistically significant.

## 5. Conclusions

The present study shows that chronic exposure to a low BPA dose could increase the risk for disease load in adult life as a consequence of higher BPA circulating levels and accumulation in adipose tissue during the neonatal period. In the same cohort of animals used in this study, impairment of immune surveillance functions in the brain of lactating animals [20], the defective first round of spermatogenesis [37], and altered liver composition in adult male rats [70] have been reported. In this respect, the effects on cerebral ventricle enlargement observed here over the treatment may affect long-term brain homeostasis and physiology. Similarly, the occurrence of mild to moderate liver micro-vesicular steatosis, in parallel to the decreased levels of the epigenetic biomarker Sirt1 in weaned animals only, point out the need for long-term monitoring of the neurological and metabolic health status of the exposed animals. Possible epigenetic mechanisms occurring via *Sirt1-AS LncRNA* and *Hdac1* may be suggested in the liver.

In conclusion, the lactating phase represents the main window of vulnerability for BPA dependent developmental perturbations in physiological processes with possible long-term consequences for health status.

## Figures and Tables

**Table 1:** Experimental groups and body weight.

		*n*. of Subjects		Weight (g) Mean ± SE	
	Treatment	Males	Females	Males	Females
Lactating	Control	6	5	34 ± 4	35 ± 4
	BPA	7	6	29 ± 4	27 ± 4
Weaned	Control	9	7	220 ± 14	155 ± 10
	BPA	10	9	232 ± 11	150 ± 12

Data are expressed as mean ± SE.

**Table 2:** Histological features of liver from control and BPA-treated rats at 10–17 PND and 45–60 PND.

		Macrovesicular Steatosis	Microvesicular Steatosis	Hypertrophy	Inflammation
Lactating	Control	0.09 ± 0.10	* 0.45 ± 0.22 (*p* < 0.05)	0.00 ± 0.00	0.18 ± 0.13
	BPA	0.00 ± 0.00	1.47 ± 0.26	0.33 ± 0.13	0.13 ± 0.09
Young	Control	0.00 ± 0.00	1.00 ± 0.35	0.00 ± 0.00	0.33 ± 0.18
	BPA	0.00 ± 0.00	1.53 ± 0.23	0.26 ± 0.11	0.00 ± 0.00

Data are expressed as mean ± SE. * Statistically significant difference vs. control group.

**Table 3:** Expression analysis of inflammation biomarkers in lactating (17 PND) and weaned (60 PND) animals.

	17 PND		60 PND	
mRNA	Control (*n* = 4–6)	BPA (*n* = 4–6)	Control (*n* = 4–6)	BPA (*n* = 4–6)
*Tnfα*	1.031 ± 0.29	16.76 ± 1.071 * (*p* = 0.042)	1.026 ± 0.27	2.54 ± 1.09 * (*p* = 0.0043)
*Il-6*	1.079 ± 0.62	14.99 ± 5.6 * (*p* = 0.041)	1.094 ± 0.48	1.071 ± 0.98 (*p* = 0.87)
*Nf-kb*	1.15 ± 0,30	10,68 ± 4.1 * (*p* = 0.043)	1.19 ± 0.4	2.31 ± 0.93 * (*p* = 0.012)

Data are expressed as normalised fold expression ± SD. * Statistically significant difference vs. control group.

**Table 4:** Expression analysis of epigenetic biomarkers in weaned (60 PND) animals.

mRNA	60 PND Females		60 PND Males	
	Control (*n* = 3)	BPA (*n* = 3)	Control (*n* = 3)	BPA (*n* = 3)
*Dnmt1*	1.110 ± 0.65	0.416 ± 0.36 (*p* = 0.181)	1.164 ± 0.73	0.834 ± 0.42 (*p* = 0.359)
*Dnmt3a*	1.145 ± 0.768	0.601 ± 0.57 (*p* = 0.387)	1.039 ± 0.52	1.049 ± 0.35 (*p* = 0.985)
*Hdac1*	1.177 ± 0.25	4.477 ± 0.75* (*p* = 0.002)	1.112 ± 0.35	1.823 ± 0.15 * (*p* = 0.019)
*Hdac2*	1.16 ± 0.64	0.866 ± 0.18 (*p* = 0.483)	1.05 ± 0.41	1.56 ± 0.22 (*p* = 0.332)

Data are expressed as normalised fold expression ± SD. * Statistically significant difference vs. control group.

**Table 5:** Expression analysis of lipid and glucose metabolism biomarkers in weaned animals.

mRNA	Control (*n* = 4–6)	BPA (*n* = 4–6)
*G6Pase*	1.047 ± 0.331	1.251 ± 0.82 (*p* = 0.606)
*Star*	1.105 ± 0.299	0.809 ± 0.151 (*p* = 0.426)
*LXRa*	1.077 ± 0.41	1.025 ± 0.38 (*p* = 0.828)

Data are expressed as normalised fold expression ± SD.

**Table 6:** NAFLD Scoring System for Rodent Models.

Histological Feature		Score			
		0	1	2	3
Steatosis	macrovesicular steatosis	<5%	5–33%	33–66%	>66%
	microvesicular steatosis	<5%	5–33%	33–66%	>66%
	hypertrophy	<5%	5–33%	33–66%	>66%
Inflammation	number of inflammatory foci/field	<0.5	0.5–1.0	1.0–2.0	>2.0

**Table 7:** Primers used in the study.

	Target	Forward 5′-3′	Reverse 5′-3′	
Inflammation	*Tnfα*	ACTGATGAGAGGGAGCCCAT	CTGTGCCTCAGCCTCTTCTC	qRT-PCR
	*Il-6*	AGTCTCCTCTCCGGACTTGT	AGAGACTTCCAGCCAGTTGC	qRT-PCR
	*Nf-kb*	TCACTGAGCTCCCGATCAGA	CATACGCTGACCCTAGCCTG	qRT-PCR
Epigenetics	*Sirt1*	CACCAGAAAGAACTTCACCACCAG	ACCATCAAGCCGCCTACTAATCTG	qRT-PCR
	*Sirt1-AS lncRNA*	TCACATCATGCAAATGGAAAACTAA	TAGGACCATTACTGCCAGAGGA	Strand specific PCR
	*Sirt1-AS lncRNA*	TCACATCATGCAAATGGAAAACTAA	TAGGACCATTACTGCCAGAGGA	qRT-PCR
	*Sirt1-AS lncRNA*	--	TAGGACCATTACTGCCAGAGGA	RT
	*Dnmt1*	CTTTTTGGGTGACGGCAACTC	GCTAAGGACGATGATGAGACGC	qRT-PCR
	*Dnmt3a*	CAGCGTCACACAGAAGCATATCC	GGTCCTCACTTTGCTGAACTTGG	qRT-PCR
	*Hdac1*	ATTGGAAGGGCTGATGTG	AATGCTAATGTTGGGAGG	qRT-PCR
	*Hdac2*	GCTATCCGCTTGTCTGATGCTC	CAGTTGCCCTTGATTGTGAGATTC	qRT-PCR
Metabolism	*LXRa*	AGGGCTCCAGGAAGAGATGT	AACTCCGTTGCAGAGTCAGG	qRT-PCR
	*G6Pase*	CGTCACCTGTGAGACTGGAC	ACGACATTCAAGCACCGGAA	qRT-PCR
	*Star*	GCTGGCGAACTCTATCTGGGT	GGGAGATGCCTGAGCAAAGC	qRT-PCR
Housekeeping	*Actin-β*	AGATGACCCAGATCATGTTTGAGA	ACCAGAGGCATACAGGGACAA	qRT-PCR
	*Gapdh*	TGGAGTCTACTGGCGTCTT	TGTCATATTTCTCGTGGTTG	qRT-PCR

## Data Availability

The data that support the findings of this study are available from the corresponding author upon reasonable request.
